# TLR4 Downregulation Identifies High-Risk HPV Infection and Integration in H-SIL and Squamous Cell Carcinomas of the Uterine Cervix

**DOI:** 10.3390/cimb46100670

**Published:** 2024-10-10

**Authors:** Angela Santoro, Giuseppe Angelico, Damiano Arciuolo, Giulia Scaglione, Belen Padial Urtueta, Gabriella Aquino, Noemy Starita, Maria Lina Tornesello, Rosalia Anna Rega, Maria Carmela Pedicillo, Manuel Mazzucchelli, Ilenia Sara De Stefano, Rosanna Zamparese, Giuseppina Campisi, Giorgio Mori, Gian Franco Zannoni, Giuseppe Pannone

**Affiliations:** 1 General Pathology Unit, Department of Woman and Child’s Health and Public Health Sciences, Fondazione Policlinico Universitario Agostino Gemelli IRCCS, 00168 Rome, Italy; damiano.arciuolo@policlinicogemelli.it (D.A.); giulia.scaglione@policlinicogemelli.it (G.S.); belen.padialurtueta@guest.policlinicogemelli.it (B.P.U.); gianfranco.zannoni@unicatt.it (G.F.Z.); 2 Istituto di Anatomia Patologica, Università Cattolica del Sacro Cuore, Largo A. Gemelli 8, 00168 Rome, Italy; 3 Department of Medicine and Surgery, Kore University of Enna, 94100 Enna, Italy; giuangel86@hotmail.it; 4 AORN Ospedali dei Colli, 80131 Naples, Italy; gabryaquino@gmail.com; 5 Molecular Biology and Viral Oncology Unit, Istituto Nazionale Tumori IRCCS “Fondazione G. Pascale”, 80131 Naples, Italy; n.starita@istitutotumori.na.it (N.S.); m.tornesello@istitutotumori.na.it (M.L.T.); 6 Pathology Unit, Istituto Nazionale Tumori IRCCS “Fondazione G. Pascale”, 80131 Naples, Italy; rosalia.rega@gmail.com; 7 Pathology Unit, Department of Clinical and Experimental Medicine, University of Foggia, 71122 Foggia, Italy; mariacarmela.pedicillo@unifg.it (M.C.P.); ileniadestefano@hotmail.it (I.S.D.S.); giorgio.mori@unifg.it (G.M.); giuseppe.pannone@unifg.it (G.P.); 8 Department of Medical and Surgical Sciences and Advanced Technologies “G.F. Ingrassia”, Anatomic Pathology, University of Catania, 95123 Catania, Italy; manuel.mazzucchelli@virgilio.it; 9 Legal Medicine Unit, Ascoli Piceno Hospital C-G. Mazzoni, Viale Degli Iris 13, 63100 Ascoli Piceno, Italy; rosanna.zamparese@sanita.marche.it; 10 Department of Biomedicine, Neuroscience and Advanced Diagnostics (BiND), University of Palermo, 90127 Palermo, Italy; giuseppina.campisi@unipa.it

**Keywords:** cervical cancer, HPV, p16, TLR4, immunotherapy

## Abstract

Growing scientific evidence suggests a link between the expression of toll-like receptor 4 (TLR4) and cervical cancer carcinogenesis. Specifically, a close relation between TLR4 expression and FIGO stage, lymph node metastases, and tumor size has been reported in cervical cancer. In the present study, we aimed to evaluate the relationship between TLR4 expression levels and human papillomavirus (HPV) infection and/or high-risk (hr) HPV integration status in patients with a histological diagnosis of high-grade squamous intraepithelial lesion (H-SIL), and squamous cell carcinoma (SCC) of the uterine cervix. Sixty biopsies of cervical neoplasia, comprising H-SIL (n = 20) and SCC (n = 40), were evaluated for TLR4 expression by immunohistochemistry. All samples were positive for high-risk HPV as confirmed by in situ hybridization (ISH) and broad-spectrum PCR followed by Sanger sequencing analysis. The intensity of TLR4 staining was higher in tissues negative for intraepithelial lesion or malignancy (NILM) than in H-SIL, and further reduced in SCC. Moreover, statistically significant differences have been observed in the percentage of TLR4 expression between NILM and H-SIL and between H-SIL and SCC, with higher percentages of expression in H-SIL than in SCC. Our results showed a significant downregulation of TLR4 in HPV-related H-SIL and SCC, compared to NILM. These data support the hypothesis that TLR4 expression is suppressed in HPV-driven oncogenesis.

## 1. Introduction

Cervical cancer is one of the most prevalent cancers worldwide, ranking fourth in both incidence and mortality among all gynecological malignancies [1]. Squamous cell carcinoma (SCC) is the most frequent histotype, accounting for approximately 75–90% of cervical tumors, followed by adenocarcinoma (AC), accounting for about 10–25% [1,2,3]. The squamous intraepithelial lesions (SIL) represent the precursors of invasive carcinoma and are classified in low-grade SIL (L-SIL) and high-grade SIL (H-SIL). The L-SIL, previously defined as cervical intraepithelial grade 1 (CIN1), is characterized by the presence of mild or moderate dysplasia in the lower part of the cervical lining, while H-SIL indicates the presence of moderate (CIN2), severe dysplasia and carcinoma in situ (CIN3), affecting most of the cervical lining [2].

Human papillomaviruses (HPVs) are the primary causative agents of cervical intraepithelial neoplasia and cervical cancer. Persistent infection with high-risk HPVs, most commonly genotypes 16, 18, 45, and 31, have been consistently and strongly linked to the development of cervical H-SIL and SCC [1,2,3,4,5]. The constitutive expression of E6 and E7 genes encoded by high-risk HPVs is the main driver of the multistep transformation process affecting the infected cervical cells. The E6 and E7 oncoproteins are able to inhibit p53 and pRb oncosuppressors, respectively, and to interact with numerous cell signaling factors regulating the cell cycle, genome, stability and epigenetic modifications (PMID: 31134154). The inactivation of oncosuppressors causes the overexpression of p16 in lesions and tumors associated with high-risk HPV infections. Immunohistochemical staining of p16 has been widely used as a surrogate biomarker of high-risk HPV infection in cervical H-SIL and cervical SCC [6].

Furthermore, cytokeratin 7 (CK7), a known marker of the squamous columnar junction cells, and CK19 have been found to be expressed in cervical intraepithelial neoplasia (CIN1) at risk to progress to CIN3 compared with CK7 negative lesions, suggesting their importance as predictive marker of cervical neoplasia progression [7]. In the normal cervix, CK19 stains endocervical columnar and reserve cells as well as basal layer cells of the ectocervix [8,9]. CK19 is also expressed in SIL, squamous cell carcinoma (SCC), and adenocarcinoma [8,9]. In addition, CK19 expression extends upward from the basal to superficial layer (bottom up expression) as the SIL grade increases [8,9]. The p16 stains similarly to CK19 with the severity of SIL [10]. This might be related to the function of CK19 promoting the translation of E7 mRNA into the oncoprotein, as previously discussed [11,12].

It is well established that persistent HPV infection increases the production of cytokines and chemokines which may promote cervical cancer development [7,8].

Toll-like receptors (TLRs) are transmembrane proteins that recognize endogenous and exogenous molecules associated with pathogens, known as a pathogen-associated molecular pattern (PAMP), to activate innate and adaptive immune responses [9,10]. TLRs play key roles in the production of cytokines and activation of adaptive immune responses against pathogens [9,10]. Several studies have demonstrated that the TLRs have an anti-tumor effect by eliciting the expression of inflammatory cytokines and the activation of cytotoxic T lymphocytes [9,10]. The TLR4 specifically recognizes bacterial lipopolysaccharide and several other pathogen-associated molecular patterns as well as endogenous molecules produced by tissue damage [11,12].

The expression of TLR4 also gives rise to tumor progression, driving the activation of signaling pathways leading to the production of mitogen-associated protein kinases (MAPKs), nuclear factor (NF)-κB, and inflammatory cytokines [11,12]. Indeed, increased expression of TLR4 has been shown to induce cell proliferation, migration, invasion, and survival, protecting cancer cells against apoptosis and immune surveillance [11,12,13,14]. High expression of TLR4 has also been associated with poor survival outcome of patients with solid cancers, promoting a greater risk of disease progressions and early relapse [15,16].

Different mechanisms can be defective in HPV recognition. For instance, the PRRs (pattern recognition receptors) can detect either viral RNA or DNA, and they can be associated with membranes or localized freely in the cytosol [17,18,19].

These different classes of PRRs use common pathways to convey their signals, ultimately culminating in the expression of pro-inflammatory cytokines, such as type I interferons (IFNs), and IFN-stimulated genes (ISGs), restricting infection establishment and spreading [20].

After infection, viral DNA is released into the cytosol which can be detected by the the PRR receptor IFN-inducible protein 16 (IFI16), and by toll-like receptors 4 (TLR4) and TLR9, indicating that there is a connection between the two defense mechanisms and that one supports the other to mutual advantage [21,22].

A simple SNPs on gene regulating the IFNg-1 may contribute to this failure [23,24].

Increasing scientific evidence suggests that TLR4 promotes tumor growth and facilitates the formation of a local immunosuppressive microenvironment in HPV-positive cervical carcinoma [15,16]. TLR4 expression has been correlated with tumor grade in HPV-related cervical carcinoma. Previous studies showed that TLR4 expression was higher in invasive cervical cancers than in CIN lesions and lower in normal cervical tissues [25,26,27,28]. Additionally, compared to the HPV-negative cell line C33A, there was greater TLR4 expression in the HPV-positive cervical cancer cell lines SiHa and HeLa, suggesting a role for HPV infection in TLR4 regulation [29]. A link between increased TLR4 expression and the severity of cervical lesions was also documented, such as a close relationship between TLR4 expression and FIGO stage, lymph node metastases, and tumor size has been reported in cervical cancer [25,26]. Therefore, these preliminary data suggest an interplay between HPV infection and the activation of the host TLR signaling pathway [25,26].

Persistence of HPV infection is associated with the ability of the virus to escape immune clearance [16,25,26,27,28,29]. HPV immune escape may be linked to the ability of the virus to interfere with the expression of TLRs and TLRs signaling pathways [16,25,26,27,28,29]. However, the exact relationship between TLR and HPV is still unclear.

In the present study, we aimed to evaluate whether TLR4 expression is associated with HPV infection and/or high-risk (hr) HPV integration status in patients with a histological diagnosis of H-SIL and SCC of the uterine cervix.

## 2. Materials and Methods

### 2.1. Study Population

The criteria for the inclusion of the patients in this study are the following: (i) histopathological diagnosis of cervical H-SIL or SCC; (ii) positivity for hr-HPV by in situ hybridization (ISH) for hr-HPV probe and by broad spectrum PCR.

Tissue samples available from our cohort consisted of bioptic cervical samples of 20 H-SIL and 40 SCC. The microenvironment composition in cervical carcinoma has significant clinical implications [22], being constituted by dendritic cells, stromal cells expressing estrogen receptor alpha, T-lymphocytes, in particular T-helper 17, infiltrating the tumor (TIL), and tumor associated macrophages (TAM). In this study, phlogistic infiltrate of cancer cells has been evaluated in a semiquantitative manner scoring, with 0 for immunodesert SCC, a score of 1 for SCC with focal lymphoplasmacytic and macrophage infiltration, a score of 2 for SCC with moderate phlogistic infiltration, and a score of 3 for SCC with intense and continuous infiltration surrounding tumor buds.

### 2.2. In Situ Hybridization (ISH)

A commercially available HPV ISH system was used (Ventana Inform HPV, Tucson, AZ, USA). Briefly, in situ hybridization was performed using the Benchmark plate and an indirect streptavidin–biotin method. The hybridization signals were shown with tetrazole blue and Fast Red nuclear counterstaining. The commercially available Ventana kit includes the following probes for HR-HPV: 16, 18, 31, 33, 35, 45, 51, 52, 56, 58, and 66 (INFORM HPV III Family 16 Probe; Ventana–Roche). ISH evaluation. ISH signals were evaluated on at least ten high-power fields using Olympus BX-41 optical microscope (Shinjuku-ku, Tokyo, Japan) (HPF, original magnification ×40). Integrative cases have been defined as those with prominent nuclear punctuated (discreet dot-like) signals (I). Cases with exclusive nuclear cluster signals were evaluated as episomal (E); cases showing a prevalent nuclear cluster signals along with also focal punctuated signals of integration were evaluated as mixed episomal-integrative (E-I). According to manufacturer instructions, aimed to identify the appropriate staining pattern, artifacts or non-specific findings were considered to be non-cellular.

### 2.3. HPV-DNA Detection

Genomic DNA was analyzed for the presence of HPV using nested PCR with the degenerate oligonucleotides MY09/MY11 (“outer”) in the first reaction, which allow amplification of a sequence of approximately 450 bp of all mucosal HPVs (approximately 50 genotypes), and in the second reaction, the oligonucleotide system MGP (“inner”) internal to the MY09/MY11 sequence, which amplifies a fragment of approximately 150 bp. The amplification products obtained in the second PCR reaction were subjected to electrophoresis on a 7% polyacrylamide gel and visualized by staining with ethidium bromide. The image was digitized using the Gel Doc image acquisition system (Bio-Rad, Hercules, CA, USA). This system allowed quantification of the signal for each amplification product using a densitometric scanner. The quantification results are expressed in nanograms per microliter. This analysis revealed positivity for HPV in 100% of the samples. The amplification products were sequenced with the oligonucleotide GP5+ using automatic sequencing with the ABI PRISM system (Fisher Scientific, Waltham, MA, USA). Alignment of the nucleotide sequence, obtained by automatic sequencing, with those present in the GeneBank database using the Blast program, allowed identification of the specific HPV genotype.

### 2.4. Immunohistochemistry

Here, 4 μm serial sections from formalin-fixed and paraffin-embedded blocks were cut and mounted on polilysine-coated glass slides. Immunostaining was performed by linked streptavidin–biotin horseradish peroxidase technique (LSAB-HRP). After sequential deparaffinization and hydration, the slides were treated with 0.3% H2O2 for 15 min to quench endogenous peroxidase. The slides were submerged in 10 mM citrate buffer pH 6 and heated in the microwave three times for three minutes each time: once at 650 W, once at 350 W, and once again for antigen retrieval. After heating, the sections were blocked for 60 min with 1.5% horse serum (Santa Cruz Biotechnology, Dallas, TX, USA) diluted in PBS buffer before reaction with the primary antibody (Ab). Primary mouse monoclonal anti-TLR4 antibody has been used according to manufacturer instruction (clone 76B357.1, Novus Biologicals, Toronto, ON, Canada). All the slides were treated with biotinylated species-specific secondary antibodies and streptavidin–biotin enzyme reagent, and the colour was developed by 3,3′-diaminobenzidine tetrahydrochloride using the standard automatic Ventana Benchmark System^®^ (VENTANA, San Diego, CA, USA). Sections were counterstained with Mayer’s haematoxylin and mounted using xylene-based mounting medium. For every staining, negative control slides devoid of main antibody were incorporated. The results of the immunohistochemical staining were evaluated separately by two observers. Immunostained cells were counted in at least 10 high-power fields (HPF) analyzed by a light microscope. For each case, the cumulative percentage of positive cells in all sections was determined as follows: score 0, negative staining; score 1, positive staining in <10% of neoplastic cells; score 2, positive staining in 10–50% of neoplastic cells; score 3, positive staining in 51–80% of neoplastic cells; score 4, positive staining in >80% of neoplastic cells. The following staining patterns were considered as positive staining: M (membranous staining); C (cytoplasmic staining); MC (membranous and cytoplasmic). Staining intensity was evaluated as follows: 0, negative; 1, faint; 2, moderate; 3, high. Finally, the staining intensity was multiplied by the percentage of TLR-4 positive cells to obtain the final expression score, ranging from 0 to 300 units.

All tissue samples were immunohistochemically stained with monoclonal mouse anti-human Cytokeratin 19 antibody (prediluted, A53-B/A2.26) and with mouse monoclonal anti-human p16. The immunostaining patterns were ultimately classified as negative, weak positive, moderate positive, or strong positive based on the staining intensity and proportion. p16 was evaluated according to the international standardized scoring method, as reported elsewhere [16] Double immunostaining for p16 (DAB) and TLR-4 (Fast Red) have been adopted.

## 3. Results

### 3.1. Clinicopathological Features

The study group included 60 female patients diagnosed with H-SIL (n = 20) and SCC (n = 40). The mean age of SCC patients was 51 years (range: 42–58), with 21 cases of women under the age of 50 and 19 cases of women over the age of 50. In 28, cases the neoplasm is localized only in the uterine cervix, while in 12 cases it also involves the body of the uterus. The histological grades of the cases included in our study were Grade 1 in 1 case, Grade 2 in 2 cases, Grade 3 in 34 cases, and Grade x in 3 cases (Table 1). The mean age of H-SIL patients was 36.5 years (range: 19–64).

### 3.2. HPV-DNA: ISH and PCR

All samples have shown positivity for the hr-HPV probe by in situ hybridization (ISH). The most frequently identified genotype in neoplasms was HPV16 (23 patients), followed by HPV 73 (1 case), HPV66-73 (1 case), HPV6-18 (1 case), HPV39 (1 case), HPV16-81 (1 case), HPV81 (1 case), and HPV18 (1 case).

### 3.3. Staining Intensity (SI) and Percentage and Combined Score (CS) of TLR4 IHC Expression in H-SIL and SCC

**Figure 1:**
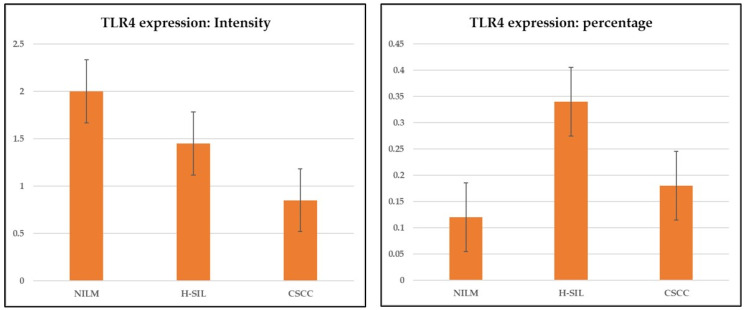
TLR4 expression intensity and percentage. The analysis of TLR4 SI in NILM, H-SIL, and SCC samples showed a statistically significant difference between the 3 groups.

Immunohistochemical staining was restricted to the cell membrane and/or the cytoplasm. No nuclear staining was identified in any of the samples. The analysis of TLR4-SI in NILM, H-SIL, and SCC samples showed a statistically significant difference between the three groups (*p* = 0.001; ANOVA) (Figure 1, Table 2). In detail, staining intensity was higher in NILM than in H-SIL, and further reduced in SCC samples.

**Figure 2:**
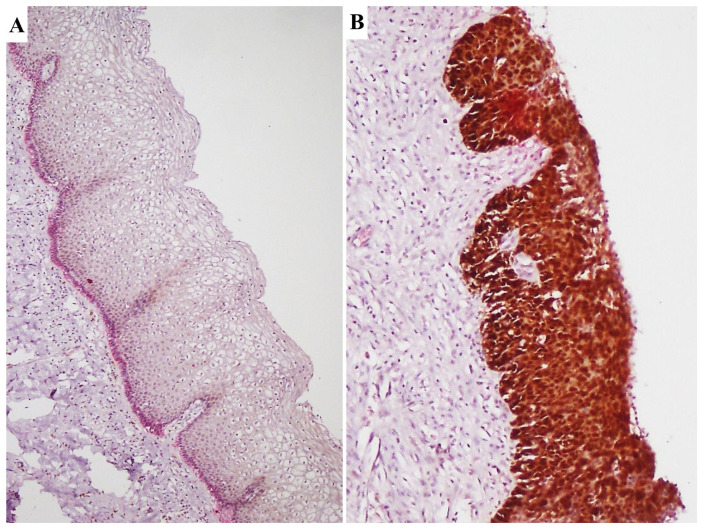
(**A**) NILM showing negative immunostaining for TLR4. (**B**) H-SIL showing diffuse and intense immunostaining for TLR4.

Moreover, statistically significant differences have been observed in the percentage of TLR4 expression between NILM and H-SIL and between H-SIL and SCC samples (*p* < 0.05; ANOVA), with higher percentages of expression in H-SIL than in SCC. Figure 2 shows NILM tissue with negative immunostaining for TLR4 and H-SIL, with diffuse and intense immunostaining for TLR4.

In HPV-positive cases, where we could evaluate the normal epithelium surrounding the tumor, we observed a stronger expression of TLR4 in the NILM epithelium (especially in the basal layer) and a downregulation of TLR4 in H-SIL and hr-HPV-integrated SCCs.

We also observed that TLR4-CS expression (IHC intensity X percentage of positive cells) is downregulated in H-SIL and in SCC samples when compared with non-neoplastic cervical tissue (*p* = 0.004; Kruskal–Wallis test).

**Figure 3:**
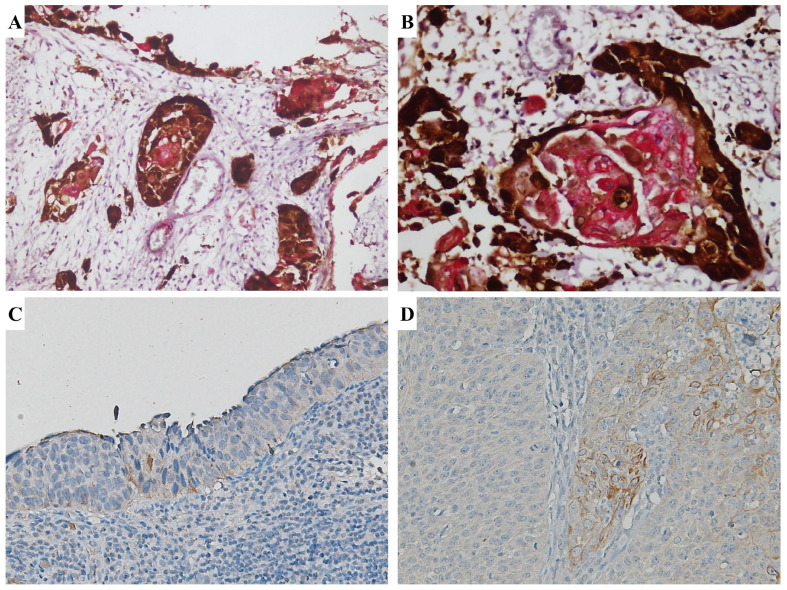
TLR4 staining patterns. (**A**,**B**) Double immunostaining for p16 (DAB–brown) and TLR-4 (Fast Red) in SCC. With double immunostaining, p16 staining was distributed at the periphery of the tumoral nests while TLR4 was more evident centrally, in more differentiated tumoral cells. (**C**) A case of H-SIL showing low TLR-4 expression (downregulation) is depicted. (**D**) A case of SCC showing moderate TLR4 expression.

Considering the study group of SCC samples, we have observed two different patterns of TLR4 expression: (1) TLR4-positive SCC samples (12 cases); (2) TLR4-negative SCC samples (28 cases). Moreover, we observed the following results:

(1) TLR4 moderately expressed SCC samples showed high levels of immune infiltrate; (2) TLR4-negative SCC samples showed scanty phlogistic cells or no TILs; (3) a lower expression of TLR4 was observed in HPV16-positive tumors when compared to other HPV genotypes related tumors; (4) using double immunostaining, a different pattern of co-distribution of p16 and TLR-4 in a subset of SCC samples has been observed, with p16 staining being distributed at the periphery of the tumoral nest and TLR4 being more evident centrally, in more differentiated tumoral cells (Figure 3).

### 3.4. Staining Intensity and Percentage of CK19 IHC Expression in H-SIL and SCC

The analysis of the intensity of the immunohistochemical expression of CK19 showed a high intensity in all samples, with an increased expression in H-SIL and SCC samples in comparison with the normal epithelium, but without a statistically significant difference (*p* > 0.05; ANOVA).

**Figure 4:**
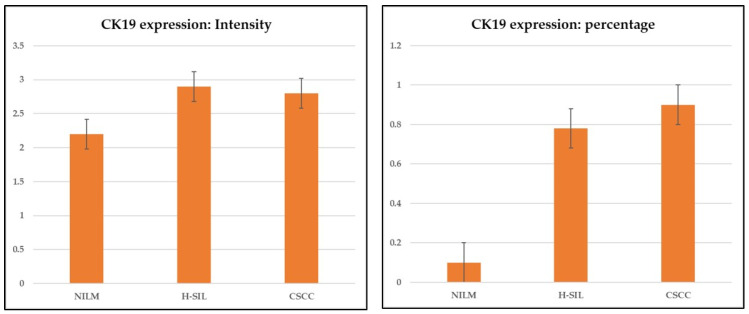
Immunohistochemical expression of CK19. The analysis of percentages of immunohistochemical expression of CK19 showed a statistically significative difference between normal epithelium, H-SIL, and SCC (*p* < 0.05; ANOVA).

The analysis of the percentage of the immunohistochemical expression of CK19, however, showed a statistically significative difference between the normal epithelium, H-SIL, and SCC samples (*p* < 0.05; ANOVA) (Figure 4, Table 3).

**Figure 5:**
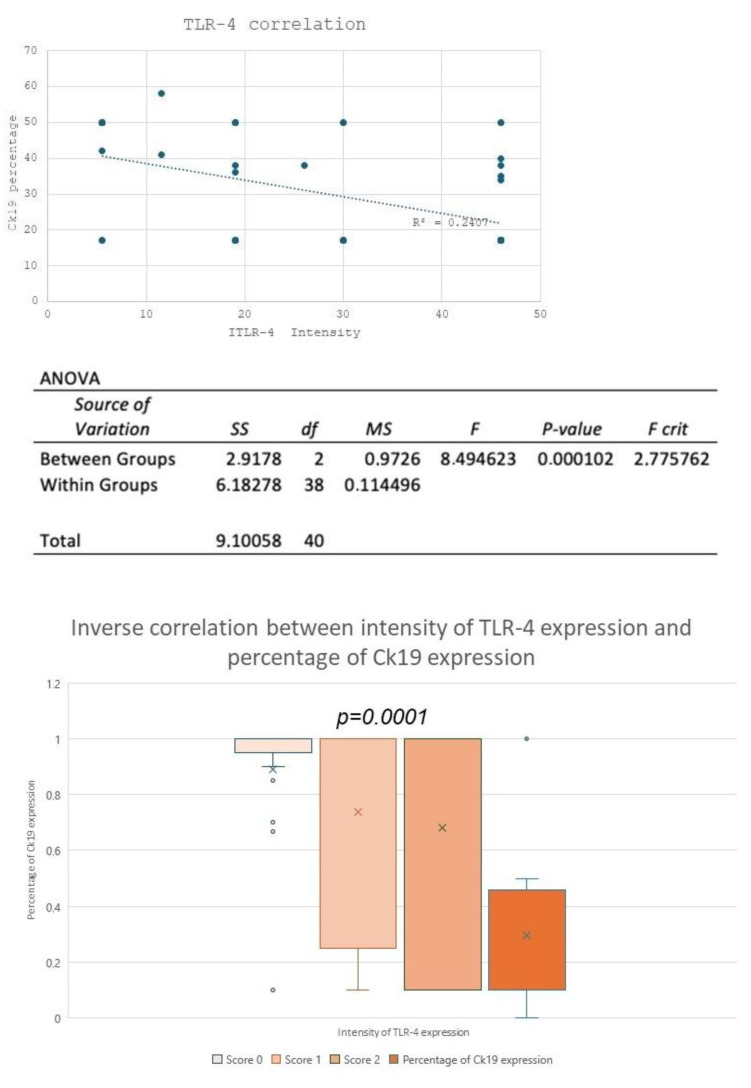
Correlation expression of TLR4 and CK19. Inverse statistically significant correlation between TLR4-SI and CK19 percentage of expressions (R = −0.4906; *p* = 0.0001, ANOVA), with CK19 upregulation and TLR4 downregulation in the cervical carcinogenetic process.

We also observed an inverse statistically significant correlation between TLR4-SI and CK19 percentage of expression (R = −0.4906; *p* = 0.0001, ANOVA), with a CK19 upregulation and a TLR4 downregulation in the cervical carcinogenetic process (Figure 5).

**Figure 6:**
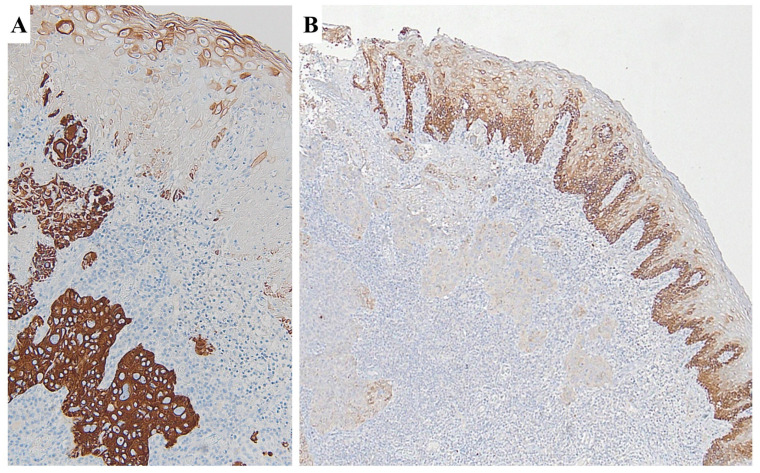
CK19 upregulation and TLR4 downregulation in the cervical carcinogenetic process. (**A**) Immunohistochemical expression of CK19 SCC; (**B**) immunohistochemical expression of TLR4 in the same case of SCC.

Figure 6 presents a case of SCC showing diffuse staining for CK19 in the invasive component and low expression in the surface epithelium. Conversely, TLR4 was moderately expressed in the surface epithelium, while low expression was observed in the invasive component. 

### 3.5. Tumor-Related Inflammation

**Figure 7:**
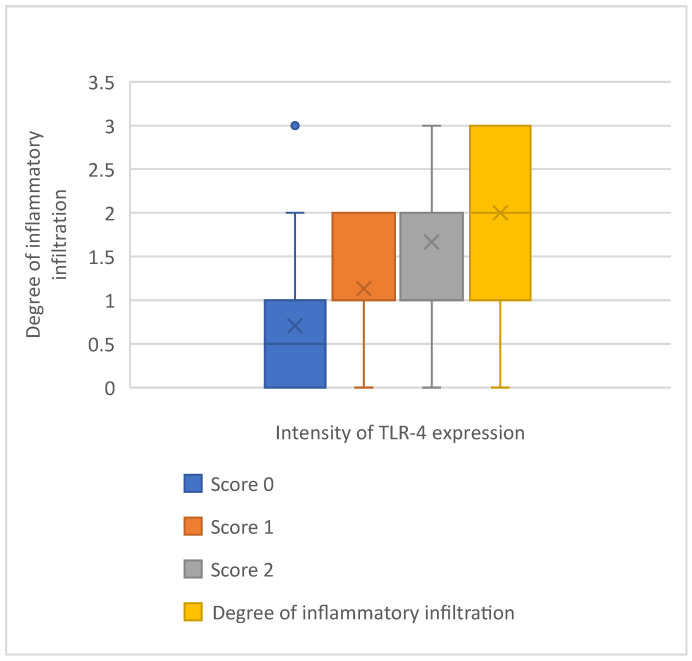
Correlation between intensity of TLR-4 expression and the degree of inflammatory infiltration. A statistically significant correlation between TLR4 downregulation and inflammatory tumoral microenvironment has been demonstrated (R = 0.5341; *p* = 0.001, ANOVA).

We have analyzed the presence and the levels of tumor-related inflammation. We assessed the inflammatory infiltration with a score ranging from 0 to 3, as follows: absence of inflammatory microenvironment (score 0), focal inflammatory infiltration (score 1), moderate inflammation (score 2), and intense peritumoral inflammation (score 3). A direct, statistically significant correlation between TLR4 downregulation and inflammatory tumoral microenvironment was demonstrated (R = 0.5341; *p* = 0.001, ANOVA) (Figure 7). * *p* < 0.001.

## 4. Discussion

Our results showed a significant downregulation of TLR4 in HPV-related H-SIL and SCC samples, compared to NILM, demonstrated by the substantial decrease in TLR4-SI in H-SIL and SCC samples compared to the non-neoplastic epithelium (*p* < 0.05, ANOVA). These data support the hypothesis that TLR4 expression is suppressed in HPV-driven oncogenesis [16,25,26,27,28,29]. This downregulation of TLR4 could be related to HPV integration status, as our samples were all hr-HPV-positive by ISH, with a nuclear pattern of integration. In particular, the downregulation starts during the precancerous phase, in the HSIL setting, when the viral load and integration levels reach their maximum levels when compared to invasive cervical carcinoma.

Furthermore, considering CK19 as an indirect marker of HPV integration, we have found that the percentage of immunohistochemical expression of CK19 instead showed a statistically significative difference between the normal epithelium, H-SIL, and SCC (34 7). Furthermore, TLR4 downregulation was found to correlate with an increased percentage of CK19 expression, limited to the basal layer in NILM, while there is a full-thickness expression in H-SIL and SCC [30].

Changes in the TLR4 signaling pathway as a consequence of viral infection have been already described in oral cancer [31].

The effects observed in cervical cancer may be related to the type of HPV, as well as different materials and techniques employed. One area of controversy surrounds the reported changes in TLR4 expression. Wang et al. observed that the HPV16-postive cervical carcinoma line SiHa showed higher expression of TLR4 than HPV18-positive HeLa; moreover, SiHa, but not HeLa, displays resistance to apoptosis following treatment with LPS, via TLR4 [32].

Previous studies performed on cervical carcinoma cell lines demonstrated a higher TLR4 expression in the HPV-positive cervical cancer cell lines SiHa and HeLa, compared with the HPV-negative cell line C33A, indicating a role for HPV infection in TLR4 regulation [28]. These findings suggest that neoplastic cells containing hr-HPV genomes have the potential to downregulate TLR4, thus promoting the evasion of the immune response. Therefore, in cervical cancer, TLRs may play an important role in HPV clearance when uterine cervix is infected.

The relationship between TLR4 and cervical carcinogenesis remains controversial, even though TLR4 is widely recognized as a significant mediator linking inflammation and tumorigenesis and is expressed in both immune cells and various malignancies [9,25,26,27,28,29,30]. Jiang et al. studied the connection between TLR4 and cervical carcinoma in vitro, finding that TLR4 promotes cell proliferation and resistance to apoptosis in HPV-related cervical carcinoma. Furthermore, pro-inflammatory cytokines, such as COX-2, iNOS, IL-6, IL-8, MIP-3α, TGF-β1, and VEGF, are upregulated in HPV-related cervical cancer cells in vivo following the activation of the TLR4/MyD88/NF-KB pathway [26].

In this way, the chronic inflammatory status of the cervix, caused by its susceptibility to microbial infection and as a result of sexual activity, together with TLR4, represent key endogenous factors able to promote an immunosuppressive microenvironment and cervical carcinoma progression [31,32,33,34]. In following studies, the same authors used a mouse xenotransplantation model to study the role of TLR4 in cervical cancer in vivo, confirming that TLR4 expression is closely related to HPV-related cervical carcinoma [25]; moreover, TLR4 promoted cervical cancer growth and altered immune microenvironment both in vitro and in vivo, favoring cervical carcinoma development and progression. Similarly, other authors have demonstrated high TLR4 expression in cervical carcinoma [35,36]. Conversely, other studies have detected low TLR4 expression, linked to histological grade, in a precancerous setting [37]. However, several studies have not analyzed the HPV genotype involved. Interestingly, a lack of or block of TLR4 and a pro-inflammatory cascade has been linked to a better treatment response and prognosis in many tumor types [38,39,40].

In our study, immunohistochemical analysis of cervical neoplastic tissues confirmed a significant lower expression of TLR4 protein in H-SIL and in a subset of SCC (all HPV16-positive) compared to tumors related to other HPV genotypes.

A limitation of such a study is that the expression level of TLR4 in different tissues by IHC cannot support the view that TLR4 plays a role in the occurrence and development of cervical cancer.

We assessed tumor inflammation by morphological evaluation of the inflammatory infiltration of surrounding tumors, and we found a statistically significant correlation between TLR4 downregulation and inflammatory infiltration in the tumor microenvironment. These results highlight a possible mechanism of TLR4-related immune escape induced by HPV, which may contribute to carcinogenesis. The supposed mechanism is based on TLR4 downregulation, which contributes to preventing the diffusion and presentation of viral antigens (antigenic spreading). In fact, TLR4 downregulation allows the body to avoid the activation of the immune response induced by LPS (ligand of TLR4) and, therefore, the inflammatory tissue response. This may lead to survival of HPV-infected epithelial cells and the absence of antigenic spreading.

Finally, dysfunction of the TLR4 signaling pathway, which thereafter leads to reduced pro-inflammatory cytokines secretion, could contribute to a better tumor prognosis, as observed in patients with HPV-related oropharyngeal SCC [34]. Polymorphisms of TLR genes have been associated with many diseases and may be a cause of TLR4 disfunction and a risk factor linked to cervical cancer [41]. Apart from TLR4 polymorphisms, dysregulation of adaptor molecules provides a further method of disrupting TLR4 function. For example, HeLa cells expresses TLR4 but not MD2, which is required for the activation of TLR4 in response to pathogen-associated molecular patterns (PAMPs) [42]. Molecules, such as CD14 and MD2, are also essential for LPS recognition and activation of the signaling pathway [36,37,38,39,40,41,42,43,44].

## 5. Conclusions

Our study highlights a new potential pathogenetic aspect of HPV-related cervical cancer development, taking into consideration the role of TLR4 as a new diagnostic, prognostic, and potentially therapeutic biomarker, in addition to the presence of HPV. The role of TLR4 has already been investigated for two decades. Previous studies showed that TLR4 expression was higher in invasive cervical cancers than in CIN lesions and that it was lower in normal cervical tissues [25,26,27,28].

TLR4 inactivation by the virus enables the evasion of immune response, leading to viral genome integration into the infected cells. Conversely, in HPV-negative cases, TLR4 hyperactivation leads to the activation of inflammatory pathways related to increasing cell proliferation, apoptosis inhibition, lymphatic and vascular neo-angiogenesis, and metastasis, favoring a “deviated” cancer-related inflammatory microenvironment.

TLR4 also represents a possible therapeutic target, due to its location on the cell surface, so this is an open research field worth exploring to identify more and more patient subgroups with the aim of a personalized and efficient therapy.

Our research gives some hints for a possible therapeutic approach with local TLR4-agonists to counterbalance TLR4 downregulation in the first phase of HPV-related carcinogenesis, thus stimulating TLR4 expression to enhance the immune response against the virus, favoring viral clearance. However, the scientific literature still lacks data regarding TLR4 as a protective molecular factor, promoting the immune response against HPV.

On the other hand, in advanced stages, the therapeutic strategy could involve a TLR4-antagonist, because, generally, a high inflammatory response increases the levels of TLR4, which is responsible for harmful events in the inflammatory immunosuppressive microenvironment, with a subsequent tumor-promoting role in HPV-related cervical cancer.

Further studies on larger series are needed to better understand the role of TLR4 in tumor evasion from the immune surveillance and to individuate anti-TLR4 vaccines.

## Figures and Tables

**Table 1:** Clinical–pathological variables of H-SIL and SCC cases and TNM stadium of SCC.

Variables	H-SIL (n = 20) n (%)	SCC (n = 40) n (%)
Mean age [range]	36.5 [19–64]	51 [42–58]
**Age**		
≤50	17 (32.6)	21 (48.6)
>50	3 (58)	19 (51.3)
**HPV status**		
HPV-positive	20 (100)	40 (100)
HPV-negative	0	0
**Tumor stage**		
Tis		1 (2.5)
T1		9 (22.5)
T1a1		5 (12.5)
T1b1-b2		14 (35.0)
T2		8 (20.0)
T3		1 (2.5)
Tx		2 (5.0)
**Tumor differentiation**		
G1		1 (2.5)
G2		2 (5.0)
G3		34 (85.0)
Unknown		3 (7.5)

**Table 2:** Differences in TLR4 expression in NILM, H-SIL, and CSCC.

TLR4 Expression: Intensity	NILM	H-SIL	CSCC
Average	2.053	1.474	0.868
Standard error	0.270	0.290	0.174
**TLR4 expression: percentage**	**NILM**	**H-SIL**	**CSCC**
Average	0.122	0.340	0.166
Standard error	0.024	0.084	0.046

**Table 3:** Differences in CK19 expression in NILM, H-SIL, and CSCC.

CK19 Expression: Intensity *	NILM	H-SIL	CSCC
Average	2.269	2.895	2.704
Standard error	0.318	0.072	0.101
**CK19 expression: percentage**	**NILM**	**H-SIL**	**CSCC**
Average	0.100	0.796	0.909
Standard error	0	0.084	0.035

* *p* < 0.001.

## Data Availability

Manuscript data are available from the corresponding author upon reasonable request.
